# Generalized geometrically convex functions and inequalities

**DOI:** 10.1186/s13660-017-1477-x

**Published:** 2017-08-29

**Authors:** Muhammad Aslam Noor, Khalida Inayat Noor, Farhat Safdar

**Affiliations:** 1 Department of Mathematics, King Saud University, Riyadh, Saudi Arabia; 2 Department of Mathematics, COMSATS Institute of Information Technology, Park Road, Islamabad, Pakistan

**Keywords:** 26D15, 26D10, 90C23, generalized convex functions, generalized geometrically convex functions, Hermite-Hadamard’s type inequalities, Hölder’s inequality

## Abstract

In this paper, we introduce and study a new class of generalized functions, called generalized geometrically convex functions. We establish several basic inequalities related to generalized geometrically convex functions. We also derive several new inequalities of the Hermite-Hadamard type for generalized geometrically convex functions. Several special cases are discussed, which can be deduced from our main results.

## Introduction

During the last few decades, the theory of convex analysis has turned into one of the most interesting and useful fields to study a wide class of problems arising in pure and applied sciences. Innovative techniques and calculations have yielded different directions for the study of convex analysis. In recent years, various inequalities for convex functions and their variant forms have been developed using novel techniques; see [1–7].

The theory of convex functions is closely related to theory of inequalities. It is well known that a function is convex, if and only if it satisfies an integral inequality, which is known as the Hermite-Hadamard inequality; see [8, 9]. Such types of integral inequalities are useful in finding the upper and lower bounds. For recent developments and applications, see [10–14].

The convex sets and convex functions have been extended and generalized in different directions using innovative ideas to study different problems in a general and unified frame work; see [15–21]. One of the most recent significant generalizations of convex functions is the *φ*-convex function, introduced by Gordji *et al.* [22]. These functions are non-convex functions. For recent developments, see [16, 22–26] and the references therein.

The main purpose of this paper is to introduce a new class of generalized convex functions, which are called generalized geometrically convex functions. We establish some new results by using the basic inequalities. We derive new Hermite-Hadamard integral inequalities for the generalized geometrically convex functions. Several special cases are considered. Our results are a significant and important refinement of well-known results for inequalities.

## Preliminaries

Let *I* be an interval in real line $\mathbb{R}$. Let $f:I\subset \mathbb{R}_{+}=(0,\infty )\rightarrow \mathbb{R} $ be a differentiable function on the interior $I^{0} $ of *I* and let $f:I=[x,y] \rightarrow \mathbb{R}$ and $\eta (\cdot ,\cdot ): \mathbb{R}\times \mathbb{R}\rightarrow \mathbb{R}$ be a continuous bifunction. Throughout this paper, we will use the following notation:

$$\mathbb{R} = (-\infty ,+\infty ), \qquad \mathbb{R}_{+} = (0,\infty ) \quad \mbox{and} \quad \mathbb{R}_{-} = (-\infty ,0). $$

### Definition 2.1

[22]

Let *I* be an interval in real line $\mathbb{R}$. A function $f:I=[x,y] \rightarrow \mathbb{R}$ is said to be generalized convex with respect to an arbitrary bifunction $\eta (\cdot ,\cdot ):\mathbb{R} \times \mathbb{R}\rightarrow \mathbb{R}$, if

$$\begin{aligned} f \bigl(tx +(1-t)y \bigr) \leq f(y)+t\eta \bigl( f(x),f(y) \bigr), \quad \forall x,y\in I, t \in [0,1]. \end{aligned}$$

If $\eta (x,y) = x-y$, then the generalized convex function reduces to a convex function.

Every convex function is a generalized convex function, but the converse is not true; see, for example, Examples 2.2 and 2.3.

### Example 2.2

[12]

For a convex function *f*, we may find another function *η* other than the function $\eta (x,y) = x-y $ such that f is generalized convex. Consider $f(x) = x^{2}$ and $\eta (x,y) = 2x+y$. Then we have

$$\begin{aligned} f \bigl(tx+(1-t)y \bigr) =& \bigl(tx+(1-t)y \bigr)^{2} \\ \leq & t x^{2}+ y^{2} +t(1-t)2xy \\ \leq & t x^{2}+ y^{2} +t(1-t) \bigl(x^{2}+y^{2} \bigr) \\ \leq & y^{2} +t \bigl(x^{2}+x^{2}+y^{2} \bigr) \\ =& y^{2} +t \bigl(2x^{2}+y^{2} \bigr) \\ =& f(y) +t\eta \bigl(f(x),f(y) \bigr), \quad \forall x,y \in \mathbb{R},t\in (0,1). \end{aligned}$$

Also the fact that $x^{2} \leq y^{2} +(2x^{2} +y^{2})$ and $y^{2} \leq y^{2}$, for all $x,y \in \mathbb{R}$ shows the correctness of the inequality for $t = 1$ and $t = 0$, respectively. This means that f is a generalized convex function. Note that the function $f (x) = x^{2}$ is generalized convex with respect to all $\eta (x,y) = ax+by$ with $a\geq 1, b \geq -1$ and $x,y \in \mathbb{R}$.

### Example 2.3

[12]

Consider $f:\mathbb{R} \rightarrow \mathbb{R}$ as

$$ f(x ) = \textstyle\begin{cases} -x & \text{if } x\geq 0, \\ x & \text{if } x< 0, \end{cases} $$

and define a bifunction $\eta =-x-y$ for all $x,y \in \mathbb{R}^{-}=(-\infty ,0)$. Then *f* is *η*-convex, but the converse is not true.

### Definition 2.4

[6]

Let $I\subset \mathbb{R}_{+}$. The set *I* is said to be a geometrically convex set, if

$$\begin{aligned} x^{t} y^{1-t} \in I, \quad \forall x, y\in I, t \in [0,1]. \end{aligned}$$

### Definition 2.5

[6]

A function $f:I\subset \mathbb{R}_{+}=(0,\infty )\rightarrow \mathbb{R} $ is said to be geometrically convex on *I*, if

$$\begin{aligned} f \bigl(y^{1-t}x^{t} \bigr)\leq (1-t)f(y) +t\bigl(f(x)\bigr), \quad \forall x, y\in I, t \in [0,1], \end{aligned}$$

where $(y^{1-t}x^{t})$ and $(1-t)f(y)+t(f(x))$ are the weighted geometric mean of two positive numbers *x* and *y* and the weighted arithmetic mean of $f(x)$ and $f(y)$, respectively.

We now introduce a new class of generalized convex functions on the geometrically convex set with respect to an arbitrary bifunction $\eta (\cdot,\cdot)$, which is called the generalized geometrically convex function.

### Definition 2.6

A function $f:I\subset \mathbb{R}_{+}=(0,\infty )\rightarrow\mathbb{R} $ is said to be generalized geometrically with respect to a bifunction $\eta (\cdot ,\cdot ):\mathbb{R}\times \mathbb{R}\rightarrow \mathbb{R}$, if

2.1 $$\begin{aligned} f \bigl(y^{1-t}x^{t} \bigr)\leq (1-t)f(y)+t\bigl(f(y)\bigr)+ \eta \bigl(f(x),f(y) \bigr),\quad \forall x, y\in I, t\in [0,1]. \end{aligned}$$

If $\eta (x,y)=x-y$, then the generalized geometrically convex functions reduce to geometrically convex functions given in Definition 2.5.

If $t=\frac{1}{2}$ in (2.1), then

2.2 $$\begin{aligned} f(\sqrt{xy})\leq f(y) +\frac{1}{2}\eta \bigl(f(x),f(y) \bigr),\quad \forall x, y\in I, t\in [0,1], \end{aligned}$$

which is called a generalized Jensen geometrically convex function.

We will use the following notations throughout this paper:

1. arithmetic mean:
  $$\begin{aligned} A(a,b)=\frac{a+b}{2}\quad \forall a, b\in \mathbb{R}_{+}, a\neq b, \end{aligned}$$
2. logarithmic mean:
  $$\begin{aligned} L(a,b) = \frac{b-a}{\log b-\log a}\quad \forall a, b\in \mathbb{R} _{+}, a \neq b, \end{aligned}$$
3. generalized logarithmic mean:
  $$ \L (a,b ) = \textstyle\begin{cases} [\frac{b^{p+1}-a^{p+1}}{(p+1)(b-a)}]^{\frac{1}{p}} & \text{if } p \neq -1,0, \\ L(a,b) & \text{if } p=-1, \\ \frac{1}{e}(\frac{b^{b}}{a^{a}})^{\frac{1}{b-a}} & \text{if } p=0. \end{cases} $$

## Main results

In this section, we derive some new Hermite-Hadamard type inequalities for generalized geometrically convex functions. We denote $I=[a,b]$, unless otherwise specified.

### Theorem 3.1

*Let* $f,g:I=[a,b]\rightarrow (0,\infty )$ *be generalized geometrically convex functions on* *I* *and* $a,b\in I$ *with* $a< b$. *Then*

$$\begin{aligned} &f(\sqrt{ab})-\frac{1}{2(\ln b-\ln a)} \int_{a}^{b}\frac{1}{x} \biggl[ \eta \biggl(f \biggl(\frac{a b}{x} \biggr),f(x) \biggr) \biggr]\,\mathrm{d}x \\ &\quad \leq \int_{a}^{b}\frac{1}{x}f(x)\,\mathrm{d}x\leq \frac{f(a)+f(b)}{2}+ \frac{1}{4}\bigl(\eta \bigl(f(a),f(b) \bigr)+\eta\bigl(f(b),f(a) \bigr)\bigr). \end{aligned}$$

### Proof

Let *f* be a generalized geometrically convex function on *I*. Then, $\forall a,b\in I$ and $t \in [0,1]$, we have

3.1 $$\begin{aligned} f \bigl(a^{t} b^{1-t} \bigr) \leq f(b)+t\eta \bigl(f(a),f(b) \bigr). \end{aligned}$$

Integrating (3.1) over *t* on $[0,1]$, we have

$$\begin{aligned} \int_{0}^{1}f \bigl(a^{t} b^{1-t} \bigr)\,\mathrm{d}t \leq & \int_{0}^{1} \bigl[f(b)+t \eta \bigl(f(a),f(b) \bigr) \bigr]\,\mathrm{d}t \\ =& \biggl[f(b)+\frac{1}{2}\eta \bigl(f(a),f(b) \bigr) \biggr]. \end{aligned}$$

Thus

$$\begin{aligned} \frac{1}{\ln b-\ln a} \int_{a}^{b}\frac{1}{x} \bigl[f(x) \bigr]\, \mathrm{d}x\leq \biggl[f(b)+ \frac{1}{2}\eta \bigl(f(a),f(b) \bigr) \biggr]. \end{aligned}$$

Using (2.2) and taking $x=(a^{t} b^{1-t})$ and $y=(a^{1-t} b^{t})$, we have

$$\begin{aligned} f(\sqrt{ab}) \leq \biggl[f \bigl( \bigl(a^{1-t} b^{t} \bigr) \bigr)+\frac{1}{2}\eta \bigl(f \bigl( \bigl(a^{t} b^{1-t} \bigr) \bigr),f \bigl( \bigl(a^{1-t} b^{t} \bigr) \bigr) \bigr) \biggr]. \end{aligned}$$

Integrating (3.2) over *t* on $[0,1]$, we have

$$\begin{aligned} f(\sqrt{ab})= \frac{1}{\ln b-\ln a} \int_{a}^{b}\frac{1}{x} \biggl[f(x)+ \frac{1}{2}\eta \biggl(f \biggl(\frac{ab}{x} \biggr),f(x) \biggr)\biggr]\,\mathrm{d}x. \end{aligned}$$

Thus

$$\begin{aligned} f(\sqrt{ab})-\frac{1}{2(\ln b-\ln a)} \int_{a}^{b}\frac{1}{x}\eta \biggl(f \biggl( \frac{a b}{x} \biggr),f(x) \biggr)\,\mathrm{d}x\leq \frac{1}{\ln b-\ln a} \int_{a}^{b} \frac{1}{x} \bigl[f(x) \bigr]\, \mathrm{d}x. \end{aligned}$$

Since *f* is a generalized geometrically convex function,

3.2 $$\begin{aligned}& f \bigl(a^{t} b^{1-t} \bigr) \leq f(b)+t\eta \bigl(f(a),f(b) \bigr), \end{aligned}$$

3.3 $$\begin{aligned}& f \bigl(a^{1-t} b^{t} \bigr) \leq f(a)+t\eta \bigl(f(b),f(a) \bigr). \end{aligned}$$

Adding (3.2) and (3.3), we have

3.4 $$\begin{aligned} f \bigl(a^{t} b^{1-t} \bigr)+f \bigl(a^{1-t} b^{t} \bigr) \leq f(a)+f(b)+t\eta \bigl(f(b),f(a) \bigr)+t \eta \bigl(f(a),f(b) \bigr). \end{aligned}$$

Integrating (3.4) over *t* on $[0,1]$, we have

$$\begin{aligned} \frac{2}{\ln b-\ln a} \int_{a}^{b}\frac{1}{x} \bigl[f(x) \bigr] \, \mathrm{d}x\leq {f(a)+f(b)}+\frac{1}{2}\bigl(\eta \bigl(f(a),f(b) \bigr)+\eta\bigl(f(b),f(a) \bigr)\bigr). \end{aligned}$$

This completes the proof. □

### Corollary 3.1

*If* $\eta (b,a)=b-a$ *in Theorem* 3.1, *then*

$$\begin{aligned} f (\sqrt{ab} )\leq \int_{a}^{b}\frac{1}{x}f(x)\,\mathrm{d}x\leq \frac{f(a)+f(b)}{2}. \end{aligned}$$

### Theorem 3.2

*Let* $f,g:I=[a,b]\rightarrow (0,\infty )$ *be generalized geometrically convex functions on* *I* *and* $a,b\in I$ *with* $a< b$. *Then*

$$\begin{aligned} \frac{1}{\ln b-\ln a} \int_{a}^{b}\frac{1}{x} \bigl[f(x)g(x) \bigr] \,\mathrm{d}x \leq \frac{1}{3}M(a,b)+\frac{1}{6}N(a,b), \end{aligned}$$

*where*

3.5 $$\begin{aligned}& M(a,b)= \bigl[ \bigl[f(b)+\eta \bigl(f(a),f(b) \bigr) \bigr] \bigl[g(b)+\eta \bigl(g(a),g(b) \bigr) \bigr]+f(b)g(b) \bigr], \end{aligned}$$

3.6 $$\begin{aligned}& N(a,b)= \bigl[f(b) \bigl[g(b)+\eta \bigl(g(a),g(b) \bigr) \bigr]+g(b) \bigl[f(b)+\eta \bigl(f(a),f(b) \bigr) \bigr] \bigr]. \end{aligned}$$

### Proof

Let *f*, *g* be generalized geometrically convex functions on *I*. Then, $\forall a,b\in I$ and $t \in [0,1]$, we have

3.7 $$\begin{aligned}& f \bigl(a^{t} b^{1-t} \bigr) \leq (1-t)f(b)+t \bigl[f(b)+\eta \bigl(f(a),f(b) \bigr) \bigr], \end{aligned}$$

3.8 $$\begin{aligned}& g \bigl(a^{t} b^{1-t} \bigr) \leq (1-t)g(b)+t \bigl[g(b)+\eta \bigl(g(a),g(b) \bigr) \bigr]. \end{aligned}$$

From (3.7) and (3.8), we have

3.9 $$\begin{aligned} & f \bigl(a^{t} b^{1-t} \bigr)g \bigl(a^{t} b^{1-t} \bigr) \\ &\quad \leq (1-t)^{2}f(b)g(b)+t(1-t) \bigl[f(b) \bigl[g(b)+\eta \bigl(g(a),g(b) \bigr) \bigr]+g(b) \bigl[f(b)+ \eta \bigl(f(a),f(b) \bigr) \bigr] \bigr] \\ &\qquad {}+t^{2} \bigl[ \bigl[f(b)+\eta \bigl(f(a),f(b) \bigr) \bigr] \bigl[g(b)+\eta \bigl(g(a),g(b) \bigr) \bigr] \bigr]. \end{aligned}$$

Integrating both sides of (3.9) over *t* on $[0,1]$, we have

$$\begin{aligned}& \int_{0}^{1}f \bigl(a^{t} b^{1-t} \bigr)g \bigl(a^{t} b^{1-t} \bigr)\,\mathrm{d}t \\& \quad \leq f(b)g(b) \int_{0}^{1}(1-t)^{2}\,\mathrm{d}t \\& \qquad {}+ \bigl[f(b) \bigl[g(b)+\eta \bigl(g(a),g(b) \bigr) \bigr]+g(b) \bigl[f(b)+ \eta \bigl(f(a),f(b) \bigr) \bigr] \bigr] \int_{0}^{1}t(1-t)\,\mathrm{d}t \\& \qquad{} + \bigl[ \bigl[f(b)+ \eta \bigl(f(a),f(b) \bigr) \bigr] \bigl[g(b)+ \eta \bigl(g(a),g(b) \bigr) \bigr] \bigr] \int_{0}^{1}t^{2}\,\mathrm{d}t \\& \quad =\frac{1}{3}f(b)g(b)+\frac{1}{6} \bigl[f(b) \bigl[g(b)+\eta \bigl(g(a),g(b) \bigr) \bigr]+g(b) \bigl[f(b)+ \eta \bigl(f(a),f(b) \bigr) \bigr] \bigr] \\& \qquad {}+ \frac{1}{3} \bigl[ \bigl[f(b)+\eta \bigl(f(a),f(b) \bigr) \bigr] \bigl[g(b)+\eta \bigl(g(a),g(b) \bigr) \bigr] \bigr] \\& \quad =\frac{1}{3}M(a,b)+\frac{1}{6}N(a,b). \end{aligned}$$

Thus

$$\begin{aligned} \frac{1}{\ln b-\ln a} \int_{a}^{b}\frac{1}{x} \bigl[f(x)g(x) \bigr] \,\mathrm{d}x \leq \frac{1}{3}M(a,b)+\frac{1}{6}N(a,b). \end{aligned}$$

This completes the proof. □

### Theorem 3.3

*Let* $f,g:I=[a,b]\rightarrow (0,\infty )$ *be generalized geometrically convex functions on* *I* *and* $a,b\in I$ *with* $a < b$. *Then*

$$\begin{aligned} \frac{1}{\ln b-\ln a} \int_{a}^{b}\frac{1}{x} \biggl[f(x)g \biggl( \frac{a b}{x} \biggr) \biggr] \,\mathrm{d}x\leq \frac{1}{6}M(a,b)+ \frac{1}{3}N(a,b), \end{aligned}$$

*where* $M(a,b)$ *and* $N(a,b)$ *are defined by* (3.5) *and* (3.6).

### Proof

Let *f*, *g* be generalized geometrically convex functions on *I*. Then, $\forall a,b\in I$ and $t \in [0,1]$, we have

3.10 $$\begin{aligned}& f \bigl(a^{t} b^{1-t} \bigr) \leq (1-t)f(b)+t \bigl[f(b)+\eta \bigl(f(a),f(b) \bigr) \bigr], \end{aligned}$$

3.11 $$\begin{aligned}& g \bigl(a^{1-t} b^{t} \bigr) \leq (1-t)g(a)+t \bigl[g(a)+\eta \bigl(g(b),g(a) \bigr) \bigr]. \end{aligned}$$

From (3.10) and (3.11), we have

3.12 $$\begin{aligned}& f \bigl(a^{t} b^{1-t} \bigr)g \bigl(a^{1-t} b^{t} \bigr) \\& \quad \leq (1-t)^{2}f(b)g(a)+t(1-t) \bigl[f(b) \bigl[g(a)+\eta \bigl(g(b),g(a) \bigr) \bigr] \\& \quad \quad {}+g(a) \bigl[f(b)+ \eta \bigl(f(a),f(b) \bigr) \bigr] \bigr] \\& \quad \quad {} +t^{2} \bigl[ \bigl[f(b)+\eta \bigl(f(a),f(b) \bigr) \bigr] \bigl[g(a)+\eta \bigl(g(b),g(a) \bigr) \bigr] \bigr]. \end{aligned}$$

Integrating both sides of (3.12) over *t* on $[0,1]$, we have

$$\begin{aligned}& \int_{0}^{1}f \bigl(a^{t} b^{1-t} \bigr)g \bigl(a^{1-t} b^{t} \bigr)\,\mathrm{d}t \\& \quad {}\leq f(b)g(a) \int_{0}^{1}(1-t)^{2}\,\mathrm{d}t \\& \quad \quad {} + \bigl[f(b) \bigl[g(a)+\eta \bigl(g(b),g(a) \bigr) \bigr]+g(a) \bigl[f(b)+ \eta \bigl(f(a),f(b) \bigr) \bigr] \bigr] \int_{0}^{1}t(1-t)\,\mathrm{d}t \\& \quad\quad {} + \bigl[ \bigl[f(b)+ \eta \bigl(f(a),f(b) \bigr) \bigr] \bigl[g(a)+ \eta \bigl(g(b),g(a) \bigr) \bigr] \bigr] \int_{0}^{1}t^{2}\,\mathrm{d}t \\& \quad = \frac{1}{3}f(b)g(a)+\frac{1}{6} \bigl[f(b) \bigl[g(a)+\eta \bigl(g(b),g(a) \bigr) \bigr]+g(a) \bigl[f(b)+ \eta \bigl(f(a),f(b) \bigr) \bigr] \bigr] \\& \quad \quad {}+ \frac{1}{3} \bigl[ \bigl[f(b)+\eta \bigl(f(a),f(b) \bigr) \bigr] \bigl[g(a)+\eta \bigl(g(b),g(a) \bigr) \bigr] \bigr] \\& \quad = \frac{1}{3}N(a,b)+\frac{1}{6}M(a,b). \end{aligned}$$

Thus

$$\begin{aligned} \frac{1}{\ln b-\ln a} \int_{a}^{b}\frac{1}{x} \biggl[f(x)g \biggl( \frac{a b}{x} \biggr) \biggr] \,\mathrm{d}x\leq \frac{1}{6}M(a,b)+ \frac{1}{3}N(a,b). \end{aligned}$$

This completes the proof. □

### Corollary 3.2

*If* $f = g$ *and* $\eta (x,y)=x-y$ *in Theorem* 3.3, *then*

$$\begin{aligned} \frac{1}{\ln b-\ln a} \int_{a}^{b}\frac{1}{x} \biggl[f(x)f \biggl( \frac{a b}{x} \biggr) \biggr] \,\mathrm{d}x\leq \frac{2}{3}f(b)f(a)+ \frac{1}{6} \bigl[f^{2}(b)+f^{2}(a) \bigr]. \end{aligned}$$

### Theorem 3.4

*Let* $f,g:I=[a,b]\rightarrow (0,\infty )$ *be generalized geometrically convex functions on* *I* *and* $a,b\in I$ *with* $a < b$. *Then*

$$\begin{aligned}& \frac{1}{\ln b-\ln a} \int_{a}^{b}\frac{1}{x} \biggl[f(x)g \biggl( \frac{a b}{x} \biggr) \biggr] \,\mathrm{d}x \\& \quad \leq \frac{1}{6} \bigl\{ \bigl[f^{2}(b)+g^{2}(a) \bigr] \\& \quad \quad {} + \bigl[ \bigl[g(a)+\eta \bigl(g(b),g(a) \bigr) \bigr]^{2}+ \bigl[f(b)+\eta \bigl(f(a),f(b) \bigr) \bigr]^{2} \bigr] \\& \quad \quad {} + \bigl[f(b) \bigl[f(b)+\eta \bigl(f(a),f(b) \bigr) \bigr]+g(a) \bigl[g(a)+ \eta \bigl(g(b),g(a) \bigr) \bigr] \bigr] \bigr\} . \end{aligned}$$

### Proof

Let *f*, *g* be generalized geometrically convex functions on *I*. Then, $\forall a,b\in I$ and $t \in [0,1]$, we have

3.13 $$\begin{aligned}& f \bigl(a^{t} b^{1-t} \bigr) \leq (1-t)f(b)+t \bigl[f(b)+\eta \bigl(f(a),f(b) \bigr) \bigr], \end{aligned}$$

3.14 $$\begin{aligned}& g \bigl(a^{1-t} b^{t} \bigr) \leq (1-t)g(a)+t \bigl[g(a)+\eta \bigl(g(b),g(a) \bigr) \bigr]. \end{aligned}$$

Now,

$$\begin{aligned}& \frac{1}{\ln b-\ln a} \int_{a}^{b}\frac{1}{x} \biggl[f(x)g \biggl( \frac{a b}{x} \biggr) \biggr] \,\mathrm{d}x \\& \quad = \int_{0}^{1} \bigl(f \bigl(a^{t} b^{1-t} \bigr) g \bigl(a^{1-t} b^{t} \bigr) \bigr) \, \mathrm{d}t \\& \quad \leq \frac{1}{2} \int_{0}^{1} \bigl[ \bigl[ f \bigl(a^{t} b^{1-t} \bigr) \bigr]^{2}+ \bigl[g \bigl(a^{1-t} b^{t} \bigr) \bigr]^{2} \bigr]\,\mathrm{d}t \\& \quad \leq \frac{1}{2} \biggl\{ \int_{0}^{1} \bigl[(1-t)f(b)+t \bigl[f(b)+\eta \bigl(f(a),f(b) \bigr) \bigr] \bigr]^{2} \\& \quad \quad {}+ \bigl[(1-t)g(a)+t \bigl[g(a)+\eta \bigl(g(b),g(a) \bigr) \bigr] \bigr]^{2} \biggr\} \,\mathrm{d}t \\& \quad = \frac{1}{2} \biggl\{ \bigl[f^{2}(b)+g^{2}(a) \bigr] \int_{0}^{1}(1-t)^{2} \,\mathrm{d}t \\& \quad \quad {}+ \bigl[ \bigl[g(a)+\eta \bigl(g(b),g(a) \bigr) \bigr]^{2}+ \bigl[f(b)+\eta \bigl(f(a),f(b) \bigr) \bigr]^{2} \bigr] \int_{0}^{1}t^{2}\,\mathrm{d}t \\& \quad \quad {}+ \bigl[ \bigl[f(b) \bigr] \bigl[f(b)+\eta \bigl(f(a),f(b) \bigr) \bigr]+ \bigl[g(a) \bigr] \bigl[g(a)+\eta \bigl(g(b),g(a) \bigr) \bigr] \bigr] \int_{0}^{1}2t(1-t)\,\mathrm{d}t \biggr\} \\& \quad = \frac{1}{6} \bigl\{ \bigl[f^{2}(b)+g^{2}(a) \bigr] + \bigl[ \bigl[g(a)+\eta \bigl(g(b),g(a) \bigr) \bigr]^{2}+ \bigl[f(b)+ \eta \bigl(f(a),f(b) \bigr) \bigr]^{2} \bigr] \\& \quad \quad {}+ \bigl[f(b) \bigl[f(b)+\eta \bigl(f(a),f(b) \bigr) \bigr]+g(a) \bigl[g(a)+ \eta \bigl(g(b),g(a) \bigr) \bigr] \bigr] \bigr\} , \end{aligned}$$

which is the required result. □

### Corollary 3.3

*If* $f=g$ *and* $\eta (b,a)=b-a$ *in Theorem* 3.4, *then*

$$\begin{aligned}& \frac{1}{\ln b-\ln a} \int_{a}^{b}\frac{1}{x} \biggl[f(x)f \biggl( \frac{a b}{x} \biggr) \biggr] \,\mathrm{d}x \\& \quad \leq \frac{1}{6} \bigl\{ 2 \bigl[f^{2}(b)+f^{2}(a) \bigr] + \bigl[f(b)f(a)+f(a)f(b) \bigr] \bigr\} . \end{aligned}$$

### Theorem 3.5

*Let* $f,g:I=[a,b]\rightarrow (0,\infty )$ *be generalized geometrically convex functions on* *I* *and* $a,b\in I$ *with* $a < b$. *Then*

$$\begin{aligned}& \frac{1}{\ln b-\ln a} \int_{a}^{b}\frac{1}{x} \biggl[f(x)g \biggl( \frac{a b}{x} \biggr) \biggr] \,\mathrm{d}x \\& \quad =\frac{1}{12} \bigl\{ \bigl[f(b)+g(a) \bigr]^{2}+ \bigl[ \bigl[f(b)+\eta \bigl(f(a),f(b) \bigr) \bigr] \bigl[g(a)+ \eta \bigl(g(b),g(a) \bigr) \bigr] \bigr]^{2} \\& \quad \quad {}+ \bigl(f(b)+g(a) \bigr) \bigl[f(b)+\eta \bigl(f(a),f(b) \bigr) \bigr] \bigl[g(a)+\eta \bigl(g(b),g(a) \bigr) \bigr] \bigr\} . \end{aligned}$$

### Proof

Let *f*, *g* be generalized geometrically convex functions on *I*. Then, $\forall a,b \in I$ and $t \in [0,1]$, we have

3.15 $$\begin{aligned}& f \bigl(a^{t} b^{1-t} \bigr) \leq (1-t)f(b)+t \bigl[f(b)+\eta \bigl(f(a),f(b) \bigr) \bigr], \end{aligned}$$

3.16 $$\begin{aligned}& g \bigl(a^{1-t} b^{t} \bigr) \leq (1-t)g(a)+t \bigl[g(a)+\eta \bigl(g(b),g(a) \bigr) \bigr]. \end{aligned}$$

Now,

$$\begin{aligned}& \frac{1}{\ln b-\ln a} \int_{a}^{b}\frac{1}{x} \biggl[f(x)g \biggl( \frac{a b}{x} \biggr) \biggr] \,\mathrm{d}x \\& \quad = \int_{0}^{1} \bigl(f \bigl(a^{t} b^{1-t} \bigr) g \bigl(a^{1-t} b^{t} \bigr) \bigr) \, \mathrm{d}t \\& \quad \leq \frac{1}{4} \int_{0}^{1} \bigl[ \bigl[ f \bigl(a^{t} b^{1-t} \bigr) \bigr]+ \bigl[g \bigl(a^{1-t} b ^{t} \bigr) \bigr] \bigr] ^{2}\,\mathrm{d}t \\& \quad \leq \frac{1}{4} \biggl\{ \int_{0}^{1} [(1-t)f(b)+t \bigl[f(b)+\eta \bigl(f(a),f(b) \bigr) \bigr] \\& \quad \quad {}+ \bigl[(1-t)g(a)+t \bigl[g(a)+\eta \bigl(g(b),g(a) \bigr) \bigr] \bigr]^{2} \biggr\} \,\mathrm{d}t \\& \quad = \frac{1}{4} \biggl\{ \int_{0}^{1} \bigl[(1-t) \bigl[f(b)+g(a) \bigr]+t \bigl[f(b)+\eta \bigl(f(a),f(b) \bigr) \bigr] \bigl[g(a)+ \eta \bigl(g(b),g(a) \bigr) \bigr] \bigr]^{2} \biggr\} \,\mathrm{d}t \\& \quad = \frac{1}{4} \biggl\{ \int_{0}^{1} [(1-t)^{2} \bigl[f(b)+g(a) \bigr]^{2}+t^{2} \bigl[ \bigl[f(b)+\eta \bigl(f(a),f(b) \bigr) \bigr] \bigl[g(a)+\eta \bigl(g(b),g(a) \bigr) \bigr] \bigr]^{2} \\& \quad \quad {}+2t(1-t) \bigl(f(b)+g(a) \bigr) \bigl[f(b)+\eta\bigl(f(a),f(b) \bigr) \bigr] \bigl[g(a)+\eta \bigl(g(b),g(a) \bigr) \bigr] \biggr\} \,\mathrm{d}t \\& \quad = \frac{1}{12} \bigl\{ \bigl[f(b)+g(a) \bigr]^{2}+ \bigl[ \bigl[f(b)+\eta \bigl(f(a),f(b) \bigr) \bigr] \bigl[g(a)+ \eta \bigl(g(b),g(a) \bigr) \bigr] \bigr]^{2} \\& \quad \quad {}+ \bigl(f(b)+g(a) \bigr) \bigl[f(b)+\eta \bigl(f(a),f(b) \bigr) \bigr] \bigl[g(a)+\eta \bigl(g(b),g(a) \bigr) \bigr] \bigr\} , \end{aligned}$$

which is the required result. □

### Corollary 3.4

*If* $f=g$ *and* $\eta (b,a)=b-a$ *in Theorem* 3.5, *then*

$$\begin{aligned}& \frac{1}{\ln b-\ln a} \int_{a}^{b}\frac{1}{x} \biggl[f(x)f \biggl( \frac{a b}{x} \biggr) \biggr] \,\mathrm{d}x \\& \quad \leq \frac{1}{12} \bigl\{ \bigl[f(b)+f(a) \bigr]^{2}+ \bigl[f(a)f(b) \bigr]^{2}+ \bigl[f(a)f(b) \bigl(f(b)+f(a) \bigr) \bigr] \bigr\} . \end{aligned}$$

### Theorem 3.6

*Let* $f,g:I=[a,b]\rightarrow (0,\infty )$ *be generalized geometrically convex functions on* *I* *and* $a,b\in I$ *with* $a< b$. *Then*

$$\begin{aligned} &f(\sqrt{a b} ) g(\sqrt{ab} ) \\ &\quad \leq \frac{1}{\ln b-\ln a} \int_{a}^{b}\frac{1}{x} \bigl[f(x)g(x) \bigr] \,\mathrm{d}x \\ &\quad \quad {}+\frac{1}{2(\ln b-\ln a)} \int_{a}^{b}\frac{1}{x} \bigl[f(x) \bigr] \biggl[ \eta \biggl(g \biggl(\frac{ab}{x} \biggr),g(x)\biggr) \biggr] \\ &\quad \quad {}+ \bigl[g(x) \bigr] \biggl[\eta \biggl(f \biggl(\frac{ab}{x}\biggr),f(x)\biggr) \biggr]\,\mathrm{d}x \\ &\quad \quad {}+\frac{1}{4(\ln b-\ln a)} \int_{a}^{b}\frac{1}{x} \biggl[\eta (f \biggl( \frac{ab}{x} \biggr),f(x) \biggr] \biggl[ \eta \biggl(g \biggl(\frac{ab}{x}\biggr),g(x)\biggr) \biggr]\,\mathrm{d}x. \end{aligned}$$

### Proof

Let *f*, *g* be generalized geometrically convex functions on *I*. Then, $\forall a,b \in I$ and $t \in [0,1]$, we have

3.17 $$\begin{aligned}& f(\sqrt{ab} )\leq f \bigl(a^{1-t}b^{t} \bigr)+ \frac{1}{2}\eta \bigl(f \bigl(a^{t}b^{1-t} \bigr),f \bigl(a ^{1-t}b^{t} \bigr) \bigr), \end{aligned}$$

3.18 $$\begin{aligned}& g(\sqrt{ab} )\leq g \bigl(a^{1-t}b^{t} \bigr)+ \frac{1}{2}\eta \bigl(g \bigl(a^{t}b^{1-t} \bigr),g \bigl(a ^{1-t}b^{t} \bigr) \bigr). \end{aligned}$$

From (3.17) and (3.18), we have

3.19 $$\begin{aligned} &f(\sqrt{a b} ) g(\sqrt{ab} ) \\ &\quad \leq \biggl[f \bigl(a^{1-t}b^{t} \bigr)+ \frac{1}{2}\eta \bigl(f \bigl(a^{t}b^{1-t} \bigr),f \bigl(a^{1-t}b ^{t} \bigr) \bigr) \biggr] \biggl[g \bigl(a^{1-t}b^{t} \bigr)+\frac{1}{2}\eta \bigl(g \bigl(a^{t}b^{1-t} \bigr),g \bigl(a^{1-t}b ^{t} \bigr) \bigr) \biggr] \\ &\quad =f \bigl(a^{1-t}b^{t} \bigr) \bigl[g \bigl(a^{1-t}b^{t} \bigr) \bigr]+\frac{1}{2} \bigl[ \bigl[f \bigl(a^{1-t}b^{t} \bigr) \bigr] \bigl[ \eta \bigl(g \bigl(a^{t}b^{1-t} \bigr),g \bigl(a^{1-t}b^{t} \bigr) \bigr) \bigr] \\ &\quad \quad {}+ \bigl[g \bigl(a^{1-t}b^{t} \bigr) \bigr] \bigl[\eta \bigl(f \bigl(a^{t}b^{1-t} \bigr),f \bigl(a^{1-t}b^{t} \bigr) \bigr) \bigr] \bigr] \\ &\quad \quad {} +\frac{1}{4} \bigl[ \bigl[\eta \bigl(g \bigl(a^{t}b^{1-t} \bigr),g \bigl(a^{1-t}b^{t} \bigr) \bigr) \bigr] \bigl[ \eta \bigl(f \bigl(a^{t}b^{1-t} \bigr),f \bigl(a^{1-t}b^{t} \bigr) \bigr) \bigr] \bigr]. \end{aligned}$$

Integrating (3.19) over *t* on $[0,1]$, we have

$$\begin{aligned} & \int_{0}^{1}f(\sqrt{a b} ) g(\sqrt{ab} )\, \mathrm{d}t \\ &\quad \leq \int_{0}^{1}f \bigl(a^{1-t}b^{t} \bigr) \bigl[g \bigl(a^{1-t}b^{t} \bigr) \bigr]\,\mathrm{d}t \\ &\quad \quad {}+ \frac{1}{2}\int_{0}^{1} \bigl[ \bigl[f \bigl(a^{1-t}b^{t}\bigr) \bigr] \bigl[\eta \bigl(g \bigl(a^{t}b^{1-t} \bigr),g \bigl(a ^{1-t}b^{t} \bigr) \bigr) \bigr] \\ &\quad \quad {}+ \bigl[g \bigl(a^{1-t}b^{t} \bigr) \bigr] \bigl[\eta \bigl(f \bigl(a^{t}b^{1-t} \bigr),f \bigl(a^{1-t}b^{t} \bigr) \bigr) \bigr] \bigr]\,\mathrm{d}t \\ &\quad \quad {} +\frac{1}{4} \int_{0}^{1} \bigl[ \bigl[\eta \bigl(g \bigl(a^{t}b^{1-t} \bigr),g \bigl(a^{1-t}b ^{t} \bigr) \bigr) \bigr] \bigl[\eta \bigl(f \bigl(a^{t}b^{1-t} \bigr),f \bigl(a^{1-t}b^{t} \bigr) \bigr) \bigr] \bigr]\, \mathrm{d}t. \end{aligned}$$

Thus

$$\begin{aligned} &f(\sqrt{a b} ) g(\sqrt{ab} ) \\ &\quad \leq \frac{1}{\ln b-\ln a} \int_{a}^{b}\frac{1}{x} \bigl[f(x)g(x) \bigr] \,\mathrm{d}x \\ &\quad \quad {}+\frac{1}{2(\ln b-\ln a)} \int_{a}^{b}\frac{1}{x} \bigl[f(x) \bigr] \biggl[ \eta \biggl(g \biggl(\frac{ab}{x} \biggr),g(x) \biggr)\biggr] \\ &\quad \quad {}+ \bigl[g(x) \bigr] \biggl[\eta \biggl(f \biggl(\frac{ab}{x}\biggr),f(x) \biggr)\biggr]\,\mathrm{d}x \\ &\quad \quad {}+\frac{1}{4( \ln b-\ln a)} \int_{a}^{b}\frac{1}{x} \biggl[\eta \biggl(f \biggl(\frac{ab}{x} \biggr),f(x) \biggr)\biggr] \biggl[ \eta \biggl(g \biggl(\frac{ab}{x}\biggr),g(x) \biggr)\biggr]\,\mathrm{d}x. \end{aligned}$$

This completes the proof. □

### Corollary 3.5

*If* $f=g$ *and* $\eta (b,a)=b-a$ *in Theorem* 3.6, *then*

$$\begin{aligned} f(\sqrt{a b} ) f(\sqrt{ab} )\leq \frac{1}{\ln b-\ln a} \int_{a} ^{b}\frac{1}{x} \bigl[f(x)f(x) \bigr] \,\mathrm{d}x. \end{aligned}$$

### Theorem 3.7

*Let* $f,g:I=[a,b]\rightarrow (0,\infty )$ *be increasing and generalized geometrically convex functions on* *I* *and* $a,b \in I $ *with* $a < b$. *Then*

$$\begin{aligned} &\frac{1}{\ln b-\ln a} \int_{a}^{b}\frac{1}{x} \bigl[f(x) \bigr] \biggl[g(b)+\frac{1}{2} \eta \bigl(g(a),g(b) \bigr) \biggr]\,\mathrm{d}x \\ &\quad \quad {}+\frac{1}{\ln b-\ln a} \int_{a}^{b}\frac{1}{x} \bigl[g(x) \bigr] \biggl[f(b)+ \frac{1}{2}\eta \bigl(f(a),f(b) \bigr) \biggr]\,\mathrm{d}x \\ &\quad \leq \frac{1}{\ln b-\ln a} \int_{a}^{b}\frac{1}{x} \bigl[f(x) \bigr] \bigl[g(x) \bigr] \,\mathrm{d}x \\ &\quad \quad {}+ \biggl[f(b)+\frac{1}{2}\eta \bigl(f(a),f(b) \bigr) \biggr] \biggl[g(b)+\frac{1}{2}\eta \bigl(g(a),g(b) \bigr) \biggr]. \end{aligned}$$

### Proof

Let *f* and *g* be generalized geometrically convex functions on *I*. Then $\forall a,b\in I$ and $t \in [0,1]$, we have

3.20 $$\begin{aligned}& f \bigl(a^{t} b^{1-t} \bigr) \leq \bigl[f(b)+t \eta \bigl(f(a),f(b) \bigr) \bigr], \end{aligned}$$

and

3.21 $$\begin{aligned}& g \bigl(a^{t} b^{1-t} \bigr) \leq \bigl[g(b)+t \eta \bigl(g(a),g(b) \bigr) \bigr]. \end{aligned}$$

Using the inequality

3.22 $$\begin{aligned} (a-b) (c-d)\geq 0 \quad \forall a,b,c,d\in \mathbb{R},a < b , c< d, \end{aligned}$$

we have

$$\begin{aligned}& \bigl[f \bigl(a^{t} b^{1-t} \bigr) \bigr] \bigl[g(b)+t\eta \bigl(g(a),g(b) \bigr) \bigr]+ \bigl[g \bigl(a^{t} b^{1-t} \bigr) \bigr] \bigl[f(b)+t\eta \bigl(f(a),f(b) \bigr) \bigr] \\& \quad \leq f \bigl(a^{t} b^{1-t} \bigr) g \bigl(a^{t} b^{1-t} \bigr)+ \bigl[f(b)+t\eta \bigl(f(a),f(b) \bigr) \bigr] \bigl[g(b)+t \eta \bigl(g(a),g(b) \bigr) \bigr]. \end{aligned}$$

Integrating the above inequality over *t* on $[0,1]$, we have

$$\begin{aligned}& \int_{0}^{1} \bigl[f \bigl(a^{t} b^{1-t} \bigr) \bigr]\,\mathrm{d}t \int_{0}^{1} \bigl[g(b)+t\eta \bigl(g(a),g(b) \bigr) \bigr] \,\mathrm{d}t \\& \qquad {} + \int_{0}^{1} \bigl[g \bigl(a^{t} b^{1-t} \bigr) \bigr]\,\mathrm{d}t \int_{0}^{1} \bigl[f(b)+t\eta \bigl(f(a),f(b) \bigr) \bigr] \,\mathrm{d}t \\& \quad \leq \int_{0}^{1}f \bigl(a^{t} b^{1-t} \bigr)\,\mathrm{d}t \int_{0}^{1} g \bigl(a^{t} b ^{1-t} \bigr)\,\mathrm{d}t \\& \qquad {} + \int_{0}^{1} \bigl[f(b)+t\eta \bigl(f(a),f(b) \bigr) \bigr]\,\mathrm{d}t \int_{0}^{1} \bigl[g(b)+t \eta \bigl(g(a),g(b) \bigr) \bigr]\,\mathrm{d}t. \end{aligned}$$

Now after simple integration, we have

$$\begin{aligned}& \frac{1}{\ln b-\ln a} \int_{a}^{b}\frac{1}{x} \bigl[f(x) \bigr] \biggl[g(b)+\frac{1}{2} \eta \bigl(g(a),g(b) \bigr) \biggr]\,\mathrm{d}x \\& \quad \quad {}+\frac{1}{\ln b-\ln a} \int_{a}^{b}\frac{1}{x} \bigl[g(x) \bigr] \biggl[f(b)+ \frac{1}{2}\eta \bigl(f(a),f(b) \bigr) \biggr]\,\mathrm{d}x \\& \quad \leq \frac{1}{\ln b-\ln a} \int_{a}^{b}\frac{1}{x} \bigl[f(x) \bigr] \bigl[g(x) \bigr] \,\mathrm{d}x \\& \quad \quad {}+ \biggl[f(b)+\frac{1}{2}\eta \bigl(f(a),f(b) \bigr) \biggr] \biggl[g(b)+\frac{1}{2}\eta \bigl(g(a),g(b) \bigr) \biggr]. \end{aligned}$$

This completes the proof. □

### Corollary 3.6

*If* $f=g$ *and* $\eta (b,a)=b-a$ *in Theorem* 3.7, *then*

$$\begin{aligned}& \frac{2}{\ln b-\ln a} \int_{a}^{b}\frac{1}{x} \bigl[f(x) \bigr] \biggl[ \frac{f(b)+f(a)}{2} \biggr]\,\mathrm{d}x \\& \quad \leq \frac{1}{\ln b-\ln a} \int_{a}^{b}\frac{1}{x} \bigl[f(x) \bigr] \bigl[f(x) \bigr] \,\mathrm{d}x+ \bigl[f(b)+f(a) \bigr]. \end{aligned}$$

## Integral inequalities

In this section, we will use the following result to obtain our main results.

### Lemma 4.1

[27]

*Let* $f:I\subset \mathbb{R}_{+}=(0,\infty )\rightarrow \mathbb{R} $ *be a differentiable function on the interior* $I^{0} $ *of* *I*, *where* $a,b \in I$ *with* $a < b$ *and* $f^{\prime }\in L[a,b]$. *Then*

$$\begin{aligned}& b f(b)-a f(a)- \int_{a}^{b} f(x)\,\mathrm{d}x \\& \quad = \frac{(\ln b-\ln a)}{2} \biggl[ \int_{0}^{1} b^{1+t} a^{1-t}f' \bigl(b^{ \frac{1+t}{2}}a^{\frac{1-t}{2}} \bigr)\,\mathrm{d}t+ \int_{0}^{1} b^{1-t} a ^{1+t}f' \bigl(b^{\frac{1-t}{2}}a^{\frac{1+t}{2}} \bigr)\,\mathrm{d}t \biggr]. \end{aligned}$$

### Theorem 4.2

*Let* $f:I\subset \mathbb{R}_{+}=(0,\infty )\rightarrow \mathbb{R}$ *be a differentiable function on the interior* $I^{0} $ *of* *I*, *where* $a,b\in I$ *with* $a < b$ *and* $f^{\prime }\in L[a,b]$. *If* $\vert f^{\prime} \vert $ *is a generalized geometrically convex function on* $[a,b]$ *for* $q\geq 1$, *then*

$$\begin{aligned}& \biggl\vert b f(b)-a f(a)- \int_{a}^{b}f(x)\,\mathrm{d}x \biggr\vert \\& \quad \leq\frac{(b-a)^{1-\frac{1}{q}}}{(2)^{\frac{1}{q}+1}} \bigl\{ b \bigl[ \bigl(2b-a-L(a,b) \bigr) \bigl\vert f^{\prime }(b) \bigr\vert ^{q} \\& \quad \quad {}+{ \bigl(L(a,b)-a \bigr)} \bigl\vert f'(b)+\eta \bigl(f'(a),f'(b)\bigr) \bigr\vert ^{q} \bigr]^{\frac{1}{q}} \\& \quad \quad {}+a \bigl[ \bigl(b-2a+L(a,b) \bigr) \bigl\vert f'(b)+\eta \bigl(f'(a),f'(b)\bigr) \bigr\vert ^{q}+ \bigl({b-L(a,b)} \bigr) \bigl\vert f'(b) \bigr\vert ^{q} \bigr]^{ \frac{1}{q}} \bigr\} . \end{aligned}$$

### Proof

Using Lemma 4.1 and Hölder’s inequality, we have

4.1 $$\begin{aligned} & \biggl\vert b f(b)-a f(a)- \int_{a}^{b}f(x)\,\mathrm{d}x \biggr\vert \\ &\quad =\frac{ab(\ln b-\ln a)}{2} \biggl[ \int_{0}^{1} \biggl(\frac{b}{a} \biggr)^{t} \bigl\vert f' \bigl(b^{\frac{1+t}{2}}a^{\frac{1-t}{2}} \bigr) \bigr\vert \,\mathrm{d}t + \int_{0}^{1} \biggl( \frac{a}{b} \biggr)^{t} \bigl\vert f' \bigl(b^{\frac{1-t}{2}}a^{\frac{1+t}{2}} \bigr) \bigr\vert \,\mathrm{d}t \biggr] \\ &\quad \leq \frac{ab(\ln b-\ln a)}{2} \biggl\{ \biggl[ \int_{0}^{1} \biggl( \frac{b}{a} \biggr)^{t}\,\mathrm{d}t \biggr]^{1-\frac{1}{q}} \biggl[ \int_{0}^{1} \biggl( \frac{b}{a} \biggr)^{t} \bigl\vert f' \bigl(b^{\frac{1+t}{2}}a^{\frac{1-t}{2}} \bigr) \bigr\vert ^{q} \,\mathrm{d}t \biggr]^{\frac{1}{q}} \\ &\quad \quad {}+ \biggl[ \int_{0}^{1} \biggl(\frac{a}{b} \biggr)^{t}\,\mathrm{d}t \biggr]^{1- \frac{1}{q}} \biggl[ \int_{0}^{1} \biggl(\frac{a}{b} \biggr)^{t} \bigl\vert f' \bigl(b^{\frac{1-t}{2}}a^{\frac{1+t}{2}} \bigr) \bigr\vert ^{q}\,\mathrm{d}t \biggr]^{\frac{1}{q}} \biggr\} . \end{aligned}$$

Now consider

4.2 $$\begin{aligned} I_{1} =& \int_{0}^{1} \biggl(\frac{b}{a} \biggr)^{t} \bigl\vert f' \bigl(b^{\frac{1+t}{2}}a^{\frac{1-t}{2}} \bigr) \bigr\vert ^{q}\,\mathrm{d}t \\ \leq & \int_{0}^{1} \bigl\vert f'(b) \bigr\vert ^{q} \biggl(\frac{1+t}{2} \biggr)+ \bigl\vert f'(b)+\eta \bigl(f'(a),f'(b)\bigr) \bigr\vert ^{q} \biggl( \frac{1-t}{2} \biggr)\,\mathrm{d}t \\ =& \bigl\vert f'(b) \bigr\vert ^{q} \int_{0}^{1} \biggl(\frac{a}{b} \biggr)^{t} \biggl(\frac{1+t}{2} \biggr)\,\mathrm{d}t + \bigl\vert f'(b)+\eta \bigl(f'(a),f'(b)\bigr) \bigr\vert ^{q} \int_{0}^{1} \biggl(\frac{b}{a} \biggr)^{t} \biggl( \frac{1-t}{2} \biggr)\,\mathrm{d}t \\ =& \bigl\vert f'(b) \bigr\vert ^{q} \frac{2b-a-L(a,b)}{2a(\ln b-\ln a)}+ \bigl\vert f'(b)+\eta \bigl(f'(a),f'(b)\bigr) \bigr\vert ^{q}\frac{L(a,b)-a}{2a( \ln b-\ln a)}. \end{aligned}$$

Similarly $I_{2}$ becomes

4.3 $$\begin{aligned} & \int_{0}^{1} \biggl(\frac{a}{b} \biggr)^{t} \bigl\vert f' \bigl(b^{\frac{1-t}{2}}a^{\frac{1+t}{2}} \bigr) \bigr\vert ^{q}\,\mathrm{d}t \\ &\quad \leq \bigl\vert f'(b)+\eta \bigl(f'(a),f'(b)\bigr) \bigr\vert ^{q}\frac{b-2a+L(a,b)}{2b(\ln b- \ln a)}+ \bigl\vert f'(b) \bigr\vert ^{q}\frac{b-L(a,b)}{2b(\ln b-\ln a)}. \end{aligned}$$

Also

4.4 $$\begin{aligned} \biggl[ \int_{0}^{1} \biggl(\frac{b}{a} \biggr)^{t}\,\mathrm{d}t \biggr]^{1-\frac{1}{q}}= \biggl( \frac{b-a}{a( \ln b-\ln a)} \biggr)^{1-\frac{1}{q}}. \end{aligned}$$

Combining (4.1), (4.2), (4.3) and (4.4), we have

$$\begin{aligned} & \biggl\vert b f(b)-a f(a)- \int_{a}^{b}f(x)\,\mathrm{d}x \biggr\vert \\ &\quad \leq \frac{(b-a)^{1-\frac{1}{q}}}{(2)^{\frac{1}{q}+1}} \bigl\{ b \bigl[ \bigl(2b-a-L(a,b) \bigr) \bigl\vert f'(b) \bigr\vert ^{q} \\ &\quad \quad {}+{ \bigl(L(a,b)-a \bigr)} \bigl\vert f'(b)+\eta \bigl(f'(a),f'(b)\bigr) \bigr\vert ^{q} \bigr]^{\frac{1}{q}} \\ &\quad \quad {}+a \bigl[ \bigl(b-2a+L(a,b) \bigr) \bigl\vert f'(b)+\eta \bigl(f'(a),f'(b)\bigr) \bigr\vert ^{q}+ \bigl({b-L(a,b)} \bigr) \bigl\vert f'(b) \bigr\vert ^{q} \bigr]^{ \frac{1}{q}} \bigr\} , \end{aligned}$$

which is the required result. □

If $\eta (b,a)=b-a$, then Theorem 4.2 reduces to the following result.

### Corollary 4.1

[27]

*Let* $f:I\subset \mathbb{R}_{+}=(0,\infty )\rightarrow\mathbb{R} $ *be a differentiable function on* $I^{0}$ (*interior of* *I*), *where* $a, b \in I$ *with* $a < b$ *and* $f'\in L[a,b]$. *If* $\vert f' \vert $ *is a generalized geometrically convex function on* $[a,b]$ *for* $q\geq 1$, *then*

$$\begin{aligned} & \biggl\vert b f(b)-a f(a)- \int_{a}^{b}f(x)\,\mathrm{d}x \biggr\vert \\ &\quad \leq \frac{(b-a)^{1-\frac{1}{q}}}{(2)^{\frac{1}{q}+1}} \bigl\{ b \bigl[ \bigl(2b-a-L(a,b) \bigr) \bigl\vert f'(b) \bigr\vert ^{q}+{ \bigl(L(a,b)-a \bigr)} \bigl\vert f'(a) \bigr\vert ^{q} \bigr]^{\frac{1}{q}} \\ &\quad \quad {}+a \bigl[ \bigl(b-2a+L(a,b) \bigr) \bigl\vert f'(a) \bigr\vert ^{q}+ \bigl({b-L(a,b)} \bigr) \bigl\vert f'(b) \bigr\vert ^{q} \bigr]^{ \frac{1}{q}} \bigr\} . \end{aligned}$$

### Theorem 4.3

*Let* $f:I\subset \mathbb{R}_{+}=(0,\infty)\rightarrow \mathbb{R}$ *be a differentiable function on* $I^{0}$ (*interior of* *I*), *where* $a, b\in I$ *with* $a < b$ *and* $f'\in L[a,b]$. *If* $\vert f' \vert $ *is a generalized geometrically convex function on* $[a,b]$ *for* $q>1$, *then*

$$\begin{aligned} & \biggl\vert b f(b)-a f(a)- \int_{a}^{b}f(x)\,\mathrm{d}x \biggr\vert \\ &\quad \leq \frac{(\ln b-\ln a)}{(2)^{1+\frac{1}{q}}} \bigl[L(a^{\frac{q}{q-1}},b ^{\frac{q}{q-1}} \bigr]^{1-\frac{1}{q}} \bigl\{ b \bigl[A \bigl(3 \bigl\vert f'(b) \bigr\vert ^{q}, \bigl\vert f'(b)+\eta \bigl(f'(a),f'(b)\bigr) \bigr\vert ^{q} \bigr) \bigr]^{\frac{1}{q}} \\ &\quad \quad {}+a \bigl[A \bigl(3 \bigl\vert f'(b)+\eta \bigl(f'(a),f'(b)\bigr) \bigr\vert ^{q} \bigr),\bigl\vert f'(b) \bigr\vert ^{q} \bigr]^{ \frac{1}{q}} \bigr\} . \end{aligned}$$

### Proof

Using Lemma 4.1 and Hölder’s inequality, we have

4.5 $$\begin{aligned} & \biggl\vert b f(b)-a f(a)-\frac{1}{b-a} \int_{a}^{b}f(x)\,\mathrm{d}x \biggr\vert \\ &\quad =\frac{ab(\ln b-\ln a)}{2} \biggl[ \int_{0}^{1} \biggl(\frac{b}{a} \biggr)^{t} \bigl\vert f' \bigl(b^{\frac{1+t}{2}}a^{\frac{1-t}{2}} \bigr) \bigr\vert \,\mathrm{d}t + \int_{0}^{1} \biggl( \frac{a}{b} \biggr)^{t} \bigl\vert f' \bigl(b^{\frac{1-t}{2}}a^{\frac{1+t}{2}} \bigr) \bigr\vert \,\mathrm{d}t \biggr] \\ &\quad \leq \frac{ab(\ln b-\ln a)}{2} \biggl\{ \biggl[ \int_{0}^{1} \biggl( \frac{b}{a} \biggr)^{\frac{qt}{q-1}}\,\mathrm{d}t \biggr]^{1-\frac{1}{q}} \biggl[ \int_{0}^{1} \bigl\vert f' \bigl(b^{\frac{1+t}{2}}a^{\frac{1-t}{2}} \bigr) \bigr\vert ^{q} \, \mathrm{d}t \biggr]^{\frac{1}{q}} \\ &\quad \quad {}+ \biggl[ \int_{0}^{1} \biggl(\frac{a}{b} \biggr)^{\frac{qt}{q-1}}\,\mathrm{d}t \biggr]^{1-\frac{1}{q}} \biggl[ \int_{0}^{1} \bigl\vert f' \bigl(b^{\frac{1-t}{2}}a^{\frac{1+t}{2}} \bigr) \bigr\vert ^{q} \, \mathrm{d}t \biggr]^{\frac{1}{q}} \biggr\} . \end{aligned}$$

Now we consider

4.6 $$\begin{aligned} I_{1} =& \int_{0}^{1} \bigl\vert f' \bigl(b^{\frac{1+t}{2}}a^{\frac{1-t}{2}} \bigr) \bigr\vert ^{q} \, \mathrm{d}t \\ \leq & \int_{0}^{1} \bigl\vert f'(b) \bigr\vert ^{q} \biggl(\frac{1+t}{2} \biggr)+ \bigl\vert f'(b)+\eta \bigl(f'(a),f'(b) \bigr) \bigr\vert ^{q} \biggl( \frac{1-t}{2} \biggr)\,\mathrm{d}t \\ =& \bigl\vert f'(b) \bigr\vert ^{q} \int_{0}^{1} \biggl(\frac{1+t}{2} \biggr) \, \mathrm{d}t + \bigl\vert f'(b)+\eta \bigl(f'(a),f'(b) \bigr) \bigr\vert ^{q} \int_{0}^{1} \biggl(\frac{1-t}{2} \biggr)\, \mathrm{d}t \\ =&\frac{3}{4} \bigl\vert f'(b) \bigr\vert ^{q}+\frac{1}{4} \bigl\vert f'(b)+\eta \bigl(f'(a),f'(b) \bigr) \bigr\vert ^{q}. \end{aligned}$$

Similarly $I_{2}$ becomes

4.7 $$\begin{aligned} & \int_{0}^{1} \bigl\vert f' \bigl(b^{\frac{1-t}{2}}a^{\frac{1+t}{2}} \bigr) \bigr\vert ^{q} \, \mathrm{d}t \leq \frac{3}{4} \bigl\vert f'(b)+\eta \bigl(f'(a),f'(b)\bigr) \bigr\vert ^{q}+ \frac{1}{4} \bigl\vert f'(b) \bigr\vert ^{q}. \end{aligned}$$

Also

4.8 $$\begin{aligned} \int_{0}^{1} \biggl(\frac{b}{a} \biggr)^{\frac{qt}{q-1}}\,\mathrm{d}t ]^{1- \frac{1}{q}}= \biggl(\frac{b^{\frac{q}{q-1}}-a^{\frac{q}{q-1}}}{( \frac{q}{q-1}) a^{\frac{q}{q-1}}(\ln b-\ln a)} \biggr)^{1-\frac{1}{q}}. \end{aligned}$$

Combining (4.5), (4.6), (4.7) and (4.8), we have

$$\begin{aligned} & \biggl\vert b f(b)-a f(a)- \int_{a}^{b}f(x)\,\mathrm{d}x \biggr\vert \\ &\quad \leq \frac{(\ln b-\ln a)}{(2)^{1+\frac{1}{q}}} \bigl[L\bigl(a^{\frac{q}{q-1}},b ^{\frac{q}{q-1}}\bigr) \bigr]^{1-\frac{1}{q}} \bigl\{ b \bigl[A \bigl(3 \bigl\vert f'(b) \bigr\vert ^{q}, \bigl\vert f'(b)+\eta \bigl(f'(a),f'(b)\bigr) \bigr\vert ^{q} \bigr) \bigr]^{\frac{1}{q}} \\ &\quad \quad {}+a \bigl[A\bigl(3 \bigl\vert f'(b)+\eta \bigl(f'(a),f'(b)\bigr) \bigr\vert ^{q}\bigr), \bigl\vert f'(b) \bigr\vert ^{q} \bigr]^{ \frac{1}{q}} \bigr\} , \end{aligned}$$

which is the required result. □

If $\eta (b,a)=b-a$, then Theorem 4.3 reduces to the following result.

### Corollary 4.2

[27]

*Let* $f:I\subset \mathbb{R}_{+}=(0,\infty )\rightarrow\mathbb{R} $ *be a differentiable function on* $I^{0} $ (*interior of* *I*), *where* $a, b\in I$ *with* $a < b$ *and* $f'\in L[a,b]$. *If* $\vert f' \vert $ *is a generalized geometrically convex function on* $[a,b]$ *for* $q>1$, *then*

$$\begin{aligned} & \biggl\vert b f(b)-a f(a)- \int_{a}^{b}f(x)\,\mathrm{d}x \biggr\vert \\ &\quad \leq \frac{(\ln b-\ln a)}{(2)^{1+\frac{1}{q}}} \bigl[L\bigl(a^{\frac{q}{q-1}},b ^{\frac{q}{q-1}}\bigr)\bigr]^{1-\frac{1}{q}} \bigl\{ b \bigl[A \bigl(3 \bigl\vert f'(b) \bigr\vert ^{q}, \bigl\vert f'(a) \bigr\vert ^{q} \bigr) \bigr]^{\frac{1}{q}} \\ &\quad \quad {}+a \bigl[A \bigl(3 \bigl\vert f'(a) \bigr\vert ^{q} \bigr), \bigl\vert f'(b) \bigr\vert ^{q} \bigr]^{\frac{1}{q}} \bigr\} . \end{aligned}$$

### Theorem 4.4

*Let* $f:I\subset \mathbb{R}_{+}=(0,\infty )\rightarrow \mathbb{R}$ *be a differentiable function on* $I^{0} $ (*interior of* *I*), *where* $a,b \in I$ *with* $a < b$ *and* $f' \in L[a,b]$. *If* $\vert f' \vert $ *is a generalized geometrically convex function on* $[a,b]$ *for* $q\geq 1$, *then*

$$\begin{aligned} & \biggl\vert b f(b)-a f(a)- \int_{a}^{b}f(x)\,\mathrm{d}x \biggr\vert \\ &\quad \leq \frac{(\ln b-\ln a)^{1-\frac{1}{q}}}{2(2q)^{\frac{1}{q}}} \bigl\{ b \bigl[ \bigl({L \bigl(a^{q},b^{q} \bigr)-a^{q}} \bigr) \bigl\vert f'(b)+\eta \bigl(f'(a),f'(b) \bigr) \bigr\vert ^{q} \\ &\quad \quad {}+ \bigl(2b^{q}-a^{q}-L \bigl(a^{q},b^{q} \bigr) \bigr) \bigl\vert f'(b) \bigr\vert ^{q} \bigr]^{\frac{1}{q}} \\ &\quad \quad {}+a \bigl[ \bigl\vert f'(b)+\eta \bigl(f'(a),f'(b) \bigr) \bigr\vert ^{q} \bigl(b^{q}-2a^{q}+L \bigl(a^{q},b^{q} \bigr) \bigr) \\ &\quad \quad {}+ \bigl({b^{q}-L \bigl(a^{q},b^{q} \bigr)} \bigr) \bigl\vert f'(b) \bigr\vert \bigr]^{\frac{1}{q}} \bigr\} . \end{aligned}$$

### Proof

Using Lemma 4.1 and Hölder’s inequality, we have

4.9 $$\begin{aligned} & \biggl\vert b f(b)-a f(a)- \int_{a}^{b}f(x)\,\mathrm{d}x \biggr\vert \\ &\quad =\frac{ab(\ln b-\ln a)}{2} \biggl[ \int_{0}^{1} \biggl(\frac{b}{a} \biggr)^{t} \bigl\vert f' \bigl(b^{\frac{1+t}{2}}a^{\frac{1-t}{2}} \bigr) \bigr\vert \,\mathrm{d}t + \int_{0}^{1} \biggl( \frac{a}{b} \biggr)^{t} \bigl\vert f' \bigl(b^{\frac{1-t}{2}}a^{\frac{1+t}{2}} \bigr) \bigr\vert \,\mathrm{d}t \biggr] \\ &\quad \leq \frac{ab(\ln b-\ln a)}{2} \biggl\{ \biggl[ \int_{0}^{1} 1\,\mathrm{d}t \biggr]^{1-\frac{1}{q}} \biggl[ \int_{0}^{1} \biggl(\frac{b}{a} \biggr)^{qt} \bigl\vert f' \bigl(b^{\frac{1+t}{2}}a^{\frac{1-t}{2}} \bigr) \bigr\vert ^{q}\,\mathrm{d}t \biggr]^{\frac{1}{q}} \\ &\quad \quad {}+ \biggl[ \int_{0}^{1} 1\,\mathrm{d}t \biggr]^{1-\frac{1}{q}} \biggl[ \int _{0}^{1} \biggl(\frac{a}{b} \biggr)^{qt} \bigl\vert f' \bigl(b^{\frac{1-t}{2}}a^{\frac{1+t}{2}} \bigr) \bigr\vert ^{q} \,\mathrm{d}t \biggr]^{\frac{1}{q}} \biggr\} . \end{aligned}$$

Now consider

4.10 $$\begin{aligned} I_{1} =& \int_{0}^{1} \biggl(\frac{b}{a} \biggr)^{qt} \bigl\vert f' \bigl(b^{\frac{1+t}{2}}a^{\frac{1-t}{2}} \bigr) \bigr\vert ^{q}\,\mathrm{d}t \\ \leq & \int_{0}^{1} \biggl(\frac{b}{a} \biggr)^{qt} \biggl[ \bigl\vert f'(b) \bigr\vert ^{t} \biggl(\frac{1+t}{2} \biggr)+ \bigl\vert f'(b)+ \eta \bigl(f'(a),f'(b) \bigr) \bigr\vert ^{q} \biggl( \frac{1-t}{2} \biggr) \biggr]\,\mathrm{d}t \\ =& \bigl\vert f'(b) \bigr\vert ^{q} \int_{0}^{1} \biggl(\frac{b}{a} \biggr)^{qt} \biggl(\frac{1+t}{2} \biggr)\,\mathrm{d}t+ \bigl\vert f'(b)+\eta \bigl(f'(a),f'(b) \bigr) \bigr\vert ^{q} \int_{0}^{1} \biggl(\frac{b}{a} \biggr)^{qt} \biggl( \frac{1-t}{2} \biggr)\,\mathrm{d}t \\ =& \bigl\vert f'(b) \bigr\vert ^{q} \frac{2b^{q}-a^{q}-L(a^{q},b^{q})}{2qa^{q}(\ln b-\ln a)}+ \bigl\vert f'(b)+\eta \bigl(f'(a),f'(b) \bigr) \bigr\vert ^{q}\frac{L(a^{q},b^{q})-a^{q}}{2qa^{q}(\ln b- \ln a)}. \end{aligned}$$

Similarly $I_{2}$ becomes

4.11 $$\begin{aligned} & \int_{0}^{1} \biggl(\frac{a}{b} \biggr)^{qt} \bigl\vert f' \bigl(b^{\frac{1-t}{2}}a^{\frac{1+t}{2}} \bigr) \bigr\vert ^{q}\,\mathrm{d}t \\ &\quad \leq \bigl\vert f'(b)+\eta \bigl(f'(a),f'(b)\bigr) \bigr\vert ^{q}\frac{b^{q}-2a^{q}+L(a^{q},b^{q})}{2qb ^{q}(\ln b-\ln a)}+ \bigl\vert f'(b) \bigr\vert ^{q} \frac{b^{q}-L(a^{q},b^{q})}{2qb^{q}(\ln b-\ln a)}. \end{aligned}$$

Combining (4.9), (4.10) and (4.11), we have

$$\begin{aligned} & \biggl\vert b f(b)-a f(a)- \int_{a}^{b}f(x)\,\mathrm{d}x \biggr\vert \\ &\quad \leq \frac{(\ln b-\ln a)^{1-\frac{1}{q}}}{2(2q)^{\frac{1}{q}}} \bigl\{ b \bigl[ \bigl({L \bigl(a^{q},b^{q} \bigr)-a^{q}} \bigr) \bigl\vert f'(b)+\eta \bigl(f'(a),f'(b) \bigr) \bigr\vert ^{q} \\ &\quad \quad + \bigl({2b^{q}-a^{q}-L \bigl(a^{q},b^{q} \bigr)} \bigr) \bigl\vert f'(b) \bigr\vert ^{q} \bigr]^{\frac{1}{q}} \\ &\quad \quad {} +a \bigl[ \bigl\vert f'(b)+\eta \bigl(f'(a),f'(b) \bigr) \bigr\vert ^{q} \bigl({b^{q}-2a^{q}+L \bigl(a^{q},b^{q} \bigr)} \bigr)+ \bigl({b^{q}-L \bigl(a^{q},b^{q} \bigr)} \bigr) \bigl\vert f'(b) \bigr\vert \bigr]^{\frac{1}{q}} \bigr\} , \end{aligned}$$

which is the required result. □

If $\eta (b,a)=b-a$, then Theorem 4.4 reduces to the following result.

### Corollary 4.3

[27]

*Let* $f:I\subset \mathbb{R}_{+}=(0,\infty )\rightarrow\mathbb{R} $ *be a differentiable function on* $I^{0} $ (*interior of* *I*), *where* $a, b\in I$ *with* $a < b$ *and* $f'\in L[a,b]$. *If* $\vert f' \vert $ *is a generalized geometrically convex function on* $[a,b]$ *for* $q\geq 1$, *then*

$$\begin{aligned} & \biggl\vert b f(b)-a f(a)- \int_{a}^{b}f(x)\,\mathrm{d}x \biggr\vert \\ &\quad \leq \frac{(\ln b-\ln a)^{1-\frac{1}{q}}}{2(2q)^{\frac{1}{q}}} \bigl\{ b \bigl[ \bigl({L \bigl(a^{q},b^{q} \bigr)-a^{q}} \bigr) \bigl\vert f'(a) \bigr\vert ^{q} \\ &\quad \quad {}+ \bigl({2b^{q}-a^{q}-L \bigl(a^{q},b^{q} \bigr)} \bigr) \bigl\vert f'(b) \bigr\vert ^{q} \bigr]^{\frac{1}{q}} +a \bigl[ \bigl\vert f'(a) \bigr\vert ^{q} \bigl( {b^{q}-2a^{q}+L \bigl(a^{q},b^{q} \bigr)} \bigr) \\ &\quad \quad {}+ \bigl({b^{q}-L \bigl(a^{q},b^{q} \bigr)} \bigr) \bigl\vert f'(b) \bigr\vert \bigr]^{\frac{1}{q}} \bigr\} . \end{aligned}$$

### Theorem 4.5

*Let* $f:I\subset \mathbb{R}_{+}=(0,\infty )\rightarrow \mathbb{R}$ *be a differentiable function on* $I^{0} $ (*interior of* *I*), *where* $a, b\in I$ *with* $a < b$ *and* $f'\in L[a,b]$. *If* $\vert f' \vert $ *is a generalized geometrically convex function on* $[a,b]$ *for* $q>1$ *and* $q>p>0$, *then*

$$\begin{aligned} & \biggl\vert b f(b)-a f(a)- \int_{a}^{b}f(x)\,\mathrm{d}x \biggr\vert \\ &\quad \leq \frac{(\ln b-\ln a)}{2(2p)^{\frac{1}{q}}} \bigl[L\bigl(a^{\frac{q-p}{q-1}},b ^{\frac{q-p}{q-1}}\bigr) \bigr]^{1-\frac{1}{q}} \bigl\{ a^{1-\frac{p}{q}}b \bigl[ \bigl({L \bigl(a ^{p},b^{p} \bigr)-a^{p}} \bigr) \bigl\vert f'(b)+\eta \bigl(f'(a),f'(b)\bigr) \bigr\vert ^{q} \\ &\quad \quad {}+ \bigl({2b^{p}-a^{p}-L \bigl(a^{p},b^{p} \bigr)} \bigr) \bigl\vert f'(b) \bigr\vert ^{q} \bigr]^{\frac{1}{q}} \bigr\} \\ &\quad \quad {}+ \bigl\{ ab^{1-\frac{p}{q}} \bigl[ \bigl({b^{p}-2a^{p}+L \bigl(a^{p},b^{p} \bigr) \bigr) \bigl\vert f'(b)+\eta \bigl(f'(a),f'(b) \bigr) \bigr\vert ^{q}} \\ &\quad \quad {}+ \bigl({b^{q}-L \bigl(a^{p},b^{p} \bigr) \bigr) \bigl\vert f'(b) \bigr\vert ^{q}} \bigr]^{\frac{1}{q}} \bigr\} . \end{aligned}$$

### Proof

Using Lemma 4.1 and Hölder’s inequality, we have

4.12 $$\begin{aligned} & \biggl\vert b f(b)-a f(a)- \int_{a}^{b}f(x)\,\mathrm{d}x \biggr\vert \\ &\quad =\frac{ab(\ln b-\ln a)}{2} \biggl[ \int_{0}^{1} \biggl(\frac{b}{a} \biggr)^{t} \bigl\vert f' \bigl(b^{\frac{1+t}{2}}a^{\frac{1-t}{2}} \bigr) \bigr\vert \,\mathrm{d}t + \int_{0}^{1} \biggl( \frac{a}{b} \biggr)^{t} \bigl\vert f' \bigl(b^{\frac{1-t}{2}}a^{\frac{1+t}{2}} \bigr) \bigr\vert \,\mathrm{d}t \biggr] \\ &\quad \leq \frac{ab(\ln b-\ln a)}{2} \biggl\{ \biggl[ \int_{0}^{1} \biggl( \frac{b}{a} \biggr)^{\frac{(q-p)t}{q-1}}\,\mathrm{d}t \biggr]^{1-\frac{1}{q}} \biggl[ \int_{0}^{1} \biggl(\frac{b}{a} \biggr)^{pt} \bigl\vert f' \bigl(b^{\frac{1+t}{2}}a^{\frac{1-t}{2}} \bigr) \bigr\vert ^{q}\,\mathrm{d}t \biggr]^{\frac{1}{q}} \\ &\quad\quad {}+ \biggl[ \int_{0}^{1} \biggl(\frac{a}{b} \biggr)^{\frac{(q-p)t}{q-1}} \,\mathrm{d}t \biggr]^{1-\frac{1}{q}} \biggl[ \int_{0}^{1} \biggl(\frac{a}{b} \biggr)^{pt} \bigl\vert f' \bigl(b^{\frac{1-t}{2}}a^{\frac{1+t}{2}} \bigr) \bigr\vert ^{q}\,\mathrm{d}t \biggr]^{\frac{1}{q}} \biggr\} . \end{aligned}$$

Now consider

4.13 $$\begin{aligned} I_{1} =& \int_{0}^{1} \biggl(\frac{b}{a} \biggr)^{pt} \bigl\vert f' \bigl(b^{\frac{1+t}{2}}a^{\frac{1-t}{2}} \bigr) \bigr\vert ^{q}\,\mathrm{d}t \\ \leq & \int_{0}^{1} \biggl(\frac{b}{a}\biggr)^{pt} \biggl[ \bigl\vert f'(b) \bigr\vert ^{t} \biggl(\frac{1+t}{2} \biggr)+ \bigl\vert f'(b)+ \eta \bigl(f'(a),f'(b) \bigr) \bigr\vert ^{q} \biggl( \frac{1-t}{2} \biggr) \biggr]\,\mathrm{d}t \\ =& \bigl\vert f'(b) \bigr\vert ^{q} \int_{0}^{1} \biggl(\frac{a}{b} \biggr)^{pt} \biggl(\frac{1+t}{2} \biggr)\,\mathrm{d}t + \bigl\vert f'(b)+\eta \bigl(f'(a),f'(b)\bigr) \bigr\vert ^{q} \int_{0}^{1} \biggl(\frac{b}{a} \biggr)^{pt} \biggl( \frac{1-t}{2} \biggr)\,\mathrm{d}t \\ =& \bigl\vert f'(b) \bigr\vert ^{q} \frac{2b^{p}-a^{p}-L(a^{p},b^{p})}{2pa^{p}(\ln b-\ln a)}+ \bigl\vert f'(b)+\eta \bigl(f'(a),f'(b) \bigr) \bigr\vert ^{q} \frac{L(a^{p},b^{p})-a^{p}}{2pa^{p}(\ln b-\ln a)}. \end{aligned}$$

Similarly $I_{2}$ becomes

4.14 $$\begin{aligned} & \biggl[ \int_{0}^{1} \biggl(\frac{a}{b} \biggr)^{pt} \bigl\vert f' \bigl(b^{\frac{1-t}{2}}a^{\frac{1+t}{2}} \bigr) \bigr\vert ^{q}\,\mathrm{d}t \biggr] \\ &\quad \leq \bigl\vert f'(b)+\eta \bigl(f'(a),f'(b)\bigr) \bigr\vert ^{q}\frac{b^{p}-2a^{p}+L(a^{p},b^{p})}{2pb ^{p}(\ln b-\ln a)}+ \bigl\vert f'(b) \bigr\vert ^{q} \frac{b^{p}-L(a^{p},b^{p})}{2pb^{p}(\ln b-\ln a)}. \end{aligned}$$

Also

4.15 $$\begin{aligned} \biggl[ \int_{0}^{1} \biggl(\frac{b}{a} \biggr)^{\frac{(q-p)t}{q-1}}\,\mathrm{d}t \biggr]^{1- \frac{1}{q}}= \biggl( \frac{b^{\frac{q-p}{q-1}}-a^{\frac{q-p}{q-1}}}{( \frac{q-p}{q-1}) a^{\frac{q-p}{q-1}}(\ln b-\ln a)} \biggr)^{1- \frac{1}{q}}. \end{aligned}$$

Combining (4.12), (4.13), (4.14) and (4.15), we have

$$\begin{aligned} & \biggl\vert b f(b)-a f(a)- \int_{a}^{b}f(x)\,\mathrm{d}x \biggr\vert \\ &\quad \leq \frac{(\ln b-\ln a)}{2(2p)^{\frac{1}{q}}} \bigl[L\bigl(a^{\frac{q-p}{q-1}},b ^{\frac{q-p}{q-1}}\bigr) \bigr]^{1-\frac{1}{q}} \bigl\{ a^{1-\frac{p}{q}}b \bigl[ \bigl({L \bigl(a^{p},b^{p} \bigr)-a^{p}} \bigr) \bigl\vert f'(b)+\eta \bigl(f'(a),f'(b) \bigr) \bigr\vert ^{q} \\ &\quad \quad {}+({2b^{p}-a^{p}-L \bigl(a^{p},b^{p} \bigr)} \bigl\vert f'(b) \bigr\vert ^{q} \bigr]^{\frac{1}{q}} \bigr\} \\ &\quad \quad {}+ \bigl\{ ab^{1-\frac{p}{q}} \bigl[ \bigl({b^{p}-2a^{p}+L \bigl(a^{p},b^{p} \bigr) \bigr) \bigl\vert f'(b)+\eta \bigl(f'(a),f'(b)\bigr) \bigr\vert ^{q}} \\ &\quad \quad {}+ \bigl(b^{q}-L \bigl(a^{p},b^{p} \bigr) \bigr) \bigl\vert f'(b) \bigr\vert ^{q} \bigr]^{\frac{1}{q}} \bigr\} , \end{aligned}$$

which is the required result. □

If $\eta (b,a)=b-a$, then Theorem 4.5 reduces to the following result.

### Corollary 4.4

[27]

*Let* $f:I\subset \mathbb{R}_{+}=(0,\infty )\rightarrow \mathbb{R} $ *be a differentiable function on the interior* $I^{0} $ *of* *I*, *where* $a, b\in I$ *with* $a < b$ *and* $f'\in L[a,b]$. *If* $\vert f' \vert $ *is a generalized geometrically convex function on* $[a,b]$ *for* $q>1$ *and* $q>p>0$, *then*

$$\begin{aligned} & \biggl\vert b f(b)-a f(a)- \int_{a}^{b}f(x)\,\mathrm{d}x \biggr\vert \\ &\quad \leq \frac{(\ln b-\ln a)}{2(2p)^{\frac{1}{q}}} \bigl[L\bigl(a^{\frac{q-p}{q-1}},b ^{\frac{q-p}{q-1}}\bigr) \bigr]^{1-\frac{1}{q}} \bigl\{ a^{1-\frac{p}{q}}b \bigl[ \bigl({L \bigl(a ^{p},b^{p} \bigr)-a^{p}} \bigr) \bigl\vert f'(a) \bigr\vert ^{q} \\ &\quad \quad {}+ \bigl({2b^{p}-a^{p}-L \bigl(a^{p},b^{p} \bigr)} \bigr) \bigl\vert f'(b) \bigr\vert ^{q} \bigr]^{\frac{1}{q}} \bigr\} + \bigl\{ ab^{1-\frac{p}{q}} \bigl[ \bigl({b^{p}-2a^{p}+L \bigl(a^{p},b^{p} \bigr) \bigr) \bigl\vert f'(a) \bigr\vert ^{q}} \\ &\quad \quad {}+ \bigl({b^{q}-L \bigl(a^{p},b^{p} \bigr) \bigr) \bigl\vert f'(b) \bigr\vert ^{q}} \bigr]^{\frac{1}{q}} \bigr\} . \end{aligned}$$

## Conclusion

In this paper, we have introduced and investigated a new class of generalized functions, called generalized geometrically convex functions. Some basic inequalities related to generalized geometrically convex functions have been derived. Several new inequalities of the Hermite-Hadamard type for generalized geometrically convex functions have been established. Several special cases are discussed, which can be deduced from our main results. The techniques and ideas of this paper may stimulate further research in this dynamic field. It is an interesting problem to consider these estimates in the setting of fractional calculus of meromorphic functions; see [3, 4, 17, 28, 29] and the references therein.
